# TiO_2_ nanotube immobilised 5-lipoxygenase-mediated screening and isolation of anti-inflammatory active compounds from the leaves of *lonicera japonica* thunb

**DOI:** 10.1080/14756366.2022.2121392

**Published:** 2022-09-19

**Authors:** Jinhua Zhu, Danyang Zhou, Dandan Wu, Wei Liu, Xiuhua Liu

**Affiliations:** Henan International Joint Laboratory of Medicinal Plants Utilization, College of Chemistry and Chemical Engineering, Henan University, Kaifeng, China

**Keywords:** Inflammation, enzyme mediated isolation, TiO_2_ nanotube, HPLC-MS, 5-LOX Inhibitor

## Abstract

In this work, a highly effective separation approach mediated by 5-Lipoxygenase (5-LOX) was established for screening and isolation of anti-inflammatory ingredients from leaves of *Lonicera japonica* Thunb. (LLJT). Using 5-LOX immobilised on TiO_2_ nanotubes as a microreactor, the targeted screening was exploited by combining with HPLC-MS system. Four compounds confirmed as luteolin, luteoside, lonicerin, and isochlorogenic acid C and a fraction (M1) were screened out to be potent inhibitors of 5-LOX. Their anti-inflammatory activities were further investigated and confirmed by RAW 264.7 cells inflammation model and rat foot swelling model. Furthermore, M1 was prepared by MCI GEL CHP20P column chromatography, and further separated by Pre-HPLC. One new compound confirmed to be 5,7,3′,4′-tetrahydroxyflavone-7-O-sambubioside was first isolated from LLJT. The results provide a new method for the effective separation of active components derived from natural products.

**Highlights**
- A 5-LOX mediated separation method was established for isolation of anti-inflammatory compounds.
- An anti-inflammatory ingredient was separated by MCI GEL CHP20P column chromatography.
- One new compound was first isolated from leaves of *Lonicera japonica* Thunb.
- 5-LOX was immobilised on TiO_2_ nanotubes and exploited by combining with HPLC-MS system.
- The anti-inflammatory activity of screened components was evaluated.

## 1. Introduction

Inflammation is a complex physiological response of the body to allergy, trauma, infection, physical and chemical factors and other stimuli. It plays an important role in the pathogenesis of many human diseases, such as cancer, mental disorders, cardiovascular disease, metabolic syndrome, inflammatory bowel disease, arthritis, etc.^1–3^. The arachidonic acid (AA) metabolic pathway is the main pathway of inflammation development, and 5-lipoxygenase (5-LOX) is a key enzyme in the process of inflammatory response and it is also an important target for the research of anti-inflammatory drugs^4^^,^^5^. Enzyme inhibitor can effectively reduce the catalytic activity of the enzyme and the production of inflammatory mediators, so as to achieve the purpose of reducing inflammation or even blocking the inflammatory response. In the pathogenesis of inflammation, the over-expression of cytokines produced by macrophages can induce the occurrence of acute and chronic inflammatory diseases^6^. NO is an important inflammatory mediator and is involved in the regulation of many biological activities^7^^,^^8^. Under normal conditions, NO mainly plays a role in regulation and balance, and the over-expression of NO can stimulate superoxide anion free radicals to form peroxynitrite, thereby causing oxidative stress and promoting the occurrence and development of inflammation^9^. Therefore, NO is regarded as one of the important factors to judge the inflammatory response^10^.

*Lonicera japonica* Thunb. is a traditional Chinese medicine, and it has antibacterial, anti-inflammatory, anti-tumor, anti-viral, immune regulation and other pharmacological effects^11–15^. In recent years, many studies have shown that the chemical components in the leaves of *Lonicera japonica* Thunb. are similar to those of in flowers^16^^,^^17^. Pharmacological experiments have also shown that the leaves have the same pharmacological effects as flowers, and even better than that of flowers in antiviral, antibacterial, and antioxidant activities^18^.

The traditional method for screening anti-inflammatory drugs from natural products is to use animal/cell models or free enzyme offline screening models^19–21^. Although these screening methods are effective for certain compounds with concrete structures, they have disadvantages such as low screening efficiency, difficult separation of substrates and products, and inability to obtain structural information of active ingredients from complex mixtures, which makes it more difficult for the subsequent separations.

The technology of immobilised enzyme has emerged in the 1960s. Because of its easy control, reusability, and good stability, it has been widely used in various fields such as chemistry, environment, biology, food, and medicine^22–25^. Nanomaterials have the characteristics of small particle size, large specific surface area, easy to stably combine with enzymes, effectively improving the enzyme loading capacity and enzyme stability, and being easy to carry out surface modification or coating, so they are often used as carriers for enzymes immobilisation^26–29^. Titanium dioxide nanotubes (TNTs) have great application potential in enzyme immobilisation due to their unique nanocavity structure and high specific surface area, as well as good chemical and physical stability and good biocompatibility^30–33^. In recent years, it has been reported that chromatography coupled enzyme microreactors is used to screen enzyme inhibitors from natural products^27^^,^^34–36^. Particularly, the chromatography-mass spectrometry method applied by most researchers not only meets the requirements of targeted screening of complex samples, but also can separate and identify inhibitors and obtain their structure information. It integrates screening, separation and identification, and realises the efficient and rapid screening of active components in medicinal materials.

Therefore, in this research, combining with high performance liquid chromatography (HPLC), an enzyme-mediated method for the separation of 5-LOX inhibitors from leaves of *Lonicera japonica* Thunb. (LLJT) was established by immobilising 5-LOX on titanium dioxide nanotubes. The anti-inflammatory active components were thus identified and their anti-inflammatory activities were comprehensively evaluated.

## 2. Materials and methods

**Scheme 1.**
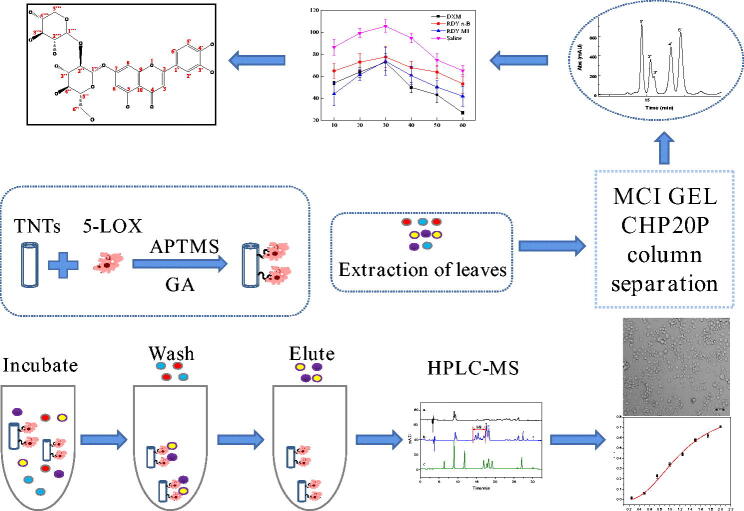
Schematic diagram of experimental procedure.

The screening and separation procedures are shown in Scheme 1. The enzyme 5-LOX was first immobilised on TNTs by glutaraldehyde, and then, the immobilised enzyme was incubated with LLJT extract, and the unimmobilised ingredients were washed away by vortex centrifugation. The compounds bound to the immobilised enzyme was eluted, and separated and identified by HPLC-MS. Their anti-inflammatory activities were further evaluated. Then, the anti-inflammatory active ingredients in the leaves were isolated by MCI GEL CHP20P column chromatography and preparative HPLC. The structures of compounds with anti-inflammatory activity were subsequently characterised and identified by mass spectrometry (MS), nuclear magnetic resonance (NMR), ultraviolet (UV) and infra-red spectroscopy (IR).

### 2.1. Chemicals and instruments

Chemicals, including neochlorogenic acid, chlorogenic acid, caffeic acid, rutin, luteoloside, lonicerin, hyperin, Isochlorogenic acid C and luteolin were purchased from Sichuan Veikeqi Biological technology Co., Ltd. (Chengdu, China). Nordihydroguaiaretic acid (NDGA), 3-aminopropyl-trimethoxysilane (APTMS), glutaraldehyde, linoleic acid (LA), and titanium dioxide nanoparticles were bought from Aladdin Biochemical Technology Co., Ltd. (Shanghai, China). Dexamethasone Acetate Injection was purchased from local drug store (Kaifeng, China). MCI GEL CHP20P high porous polymers were bought from Mitsubishi Chemical Co., Ltd. (Tokyo, Japan). Dimethyl sulfoxide (DMSO), methanol, ethanol, ethyl ether, and acetate and other chemicals were bought from Tianjin Deen Chemical Reagent Co., Ltd. (Tianjin, China). 5-LOX and endotoxin lipopolysaccharide (LPS) were purchased from Sigma-Aldrich Chemical (St. Louis, MO, USA). CCK-8 and NO kit were bought from Beyotime Biotechnology Co., Ltd. (Shanghai, China). The reagents used in the cell experiments were purchased from Zhengzhou Purcell Life Technology Co., Ltd. (Zhengzhou, China). The RAW264.7 mouse macrophage cell lines were purchased from Wuhan Prosser Life Technology Co., Ltd. (Wuhan, China).

A Bruker D8 Advance diffractometer (Bruker, Karlsruhe, Germany) using Cu Kα radiation from 5° to 60° (2θ) was used to record X-ray diffraction (XRD) data. A JEM 2100 transmission electron microscope (TEM) and a JSM-7610F scanning electron microscope (SEM) (JOEL, Peabody, MA, USA) were used to characterise the nanomaterials. The Fourier-transform infra-red (FTIR) spectra were obtained from a Bruker Vertex 70 FTIR spectrometer (Bruker Corporation, Karlsruhe, Germany). The structure of the compound was confirmed by the nuclear magnetic resonance (NMR) analyses (Bruker AV 600, GmbH, Switzerland). The UV-Vis absorption spectrum was measured by a TU-1900 spectrophotometer (Beijing, China). A (3-[4,5-dimethylthiazol-2-yl]-2,5 diphenyl tetrazolium bromide) (MTT) assay was implemented on a microplate reader (CLARIO star, BMG LABTECH, Ortenberg, Germany). A YLS-7C toe volume metre from Jinan Yiyan Technology Co., Ltd. (Jinan, China) was used. A high performance liquid chromatography (HPLC) coupled with AmaZon SL ion trap mass spectrometry (Bruker Daltonik GmbH, Bremen) was used to analyse the components in LLJT leaves extract. An Agilent 1260 HPLC system (Agilent Technologies, MA, USA) was used. Cells were observed by using an inverted fluorescence microscope (Leica DM IRBE, Deerfield, IL).

### 2.2. Preparation of 5-LOX microreactor

#### 2.2.1. Synthesis of TNTs

TNTs were synthesised by a hydrothermal method. Firstly, 1.5 g of titanium dioxide was added into 70 mL of NaOH (10 M) solution, and ultrasonically stirred for 1 h. After being evenly dispersed, it was placed in a 100 mL reaction kettle and reacted at 120 °C for 30 h. Then it was washed to neutral with hydrochloric acid (1.0 M) and deionised water, and then vacuum-dried at 110 °C to obtain titanium dioxide nanotubes. Then, 100 mg of the above nanotubes were dispersed in 40 mL of toluene solution, and 400 μL of APTMS was added, the solution was refluxed at 120 °C for 10 h. Then, the material was washed several times with anhydrous ethanol and dried under vacuum at 60 °C. The amine-functionalized TNTs (NH_2_-TNTs) were thus obtained.

#### 2.2.2. Immobilisation of 5-LOX

Glutaraldehyde (GA) was used as crosslinking agent to covalently immobilise 5-LOX on the amino terminated TNTs. The NH_2_-TNTs was added to GA solution and shaken in a incubator at room temperature (25 °C) for 8 h. The resulting product was labelled GA-TNTs. Then GA-TNTs was merged with Tris-HCl solution (0.1 M, pH 7.0) containing a certain amount of 5-LOX, and the immobilisation was performed in a shaker at 4 °C for a certain time. After that, the immobilised 5-LOX was acquired by centrifuge to remove supernatant and comprehensively rinsed with Tris-HCl buffer solution several times to remove the unimmobilised enzyme. The 5-LOX-TNTs composites were suspended in Tris-HCl solution and stored at 4 °C before use.

### 2.3. Screening procedure

The whole procedure of the proposed approach is illustrated in Scheme 1. The freshly picked leaves of *Lonicera japonica* Thunb. were washed with deionised water and then dried at 50 °C to constant weight. The dried leaves of *Lonicera japonica* Thunb. were pulverised and sieved through a 60-mesh sieve. Then the powder was degreased with petroleum ether, and reflux extracted with 80% (v/v) methanol (1:50 of solid-liquid ratio) at 80 °C for 3 h for three times. The extract solution was then combined and concentrated to obtain the crude extract.

The crude extract of leaves of *Lonicera japonica* Thunb. was added to 5% (v/v) methanol-Tris buffer solution, and dissolved by ultrasonic to prepare an incubation solution with a concentration of about 5.66 mg/mL. Then 25 mg of material immobilised with 5-LOX was combined with 1 mL of incubation solution and incubated at 25 °C for 90 min on a shaker. After incubation, the material was washed with 1 mL of buffer solution for six times. Then 1 mL of 90% (v/v) acetonitrile aqueous solution was used for ultrasonic elution for 10 min. After centrifugation, the supernatant was filtered and analysed by HPLC-MS.

The blank group was the material without enzyme immobilised, and NDGA was selected as the control group. The screening processes were the same as above.

### 2.4. Separation conditions

#### 2.4.1. Sample disposal method

The leaves of *Lonicera japonica* Thunb. were disposed as in Section 2.3 to obtain the crude extract. After that the crude extract was completely ultrasonically dissolved with deionised water, then it was subjected to fractional extraction with about three times the volume of petroleum ether, ethyl acetate and n-butanol, respectively. Each phase was concentrated under reduced pressure to obtain extracts of different polarities for following use.

#### 2.4.2. MCI Gel CHP20P column separation conditions

After the MCI GEL CHP20P column was pre-treated, the wet sample loading method was adopted. The obtained n-butanol phase sample dissolved in a volume of ultrapure water was used as the loading sample. Gradient elution was performed with ultrapure water, 10%, 20%, 30%, 40%, 50%, 60%, 80%, 100% (v/v) methanol solution, respectively, each gradient elution volume was about 3 column volume. The fractions obtained from each gradient were concentrated for following separation. After that the column was regenerated for reuse.

#### 2.4.3. HPLC-MS/MS conditions

The COSMOSIL 5C18-PAQ column (4.6 × 250 mm, 5 μm) was performed for the separation. A solution containing 0.5% (v/v) acetic acid (A) and acetonitrile (B) was used as the mobile phase. The gradient elution procedure was as follows (v/v): 12–20% B at 0–10 min, 20% B at 10–20 min, 20%-40% B at 20–25 min and 40% A at 25–30 min. The flow rate was 1.0 mL/min, the injection volume was 10 μL, the column temperature was set at 30 °C, and the detection wavelength was 327 nm. The mass spectrometry analysis was performed on the AmaZon SL series ion trap mass spectrometer with an electrospray ionisation source in the positive ion detection mode. Electronic energy was 70 ev and the full scan range was 50–800 amu. Ultrahigh pure helium and highly pure nitrogen were used as collision gas and nebulising gas, respectively. Data Analysis 4.2 software was used to collect data. The capillary voltage and the endplate offset voltage were 4.5 kV and −500 V, respectively. The atomising gas pressure was set at 15 psi, and the flow rate of dry gas was 8 L/min. The MS transfer line and ion source temperatures were set at 250 °C and 220 °C, respectively. Identification of the analytes was achieved by comparison MS spectra with standard compounds.

#### 2.4.4. Semi-preparative HPLC separation conditions

The column was a COSMOSIL 5C18-PAQ column (10 × 250 mm, 5 μm). A gradient elution of 0.5% (v/v) acetic acid (A) and acetonitrile (B) was used as follows (v/v): 12–23% B at 0–30 min, and 23–12% B at 30–35 min. The flow rate was 4.6 mL/min, the column temperature was 25 °C, the injection volume was 200 μL, and the detected wavelength was 327 nm.

## 3. Results and discussion

### 3.1. Characterisation of TNTs

**Figure 1.**
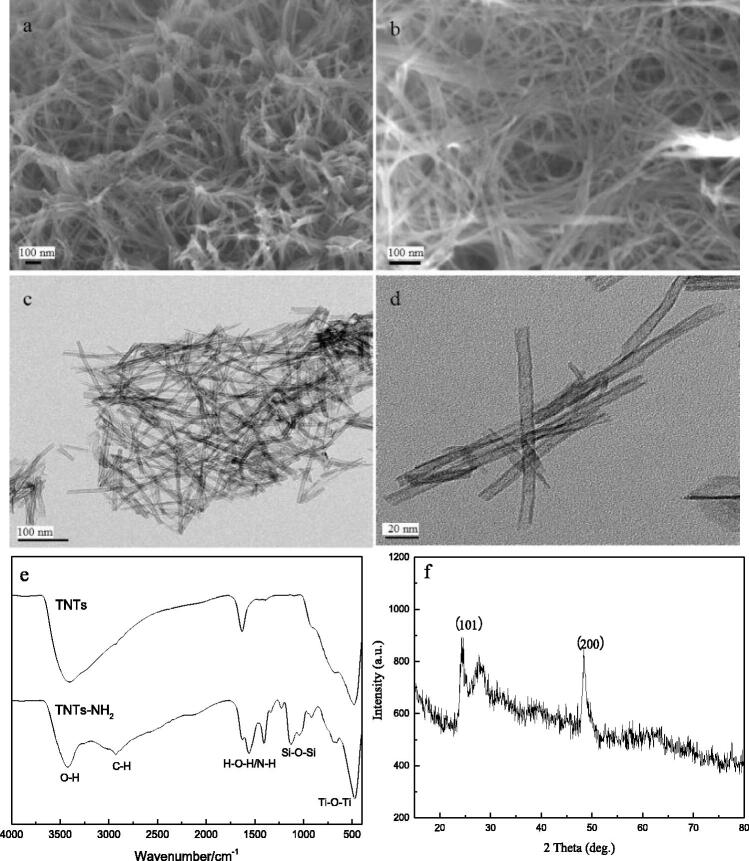
SEM (a, b), TEM (c, d), FT-IR (e) and X-ray diffraction (f) spectra of TNTs.

The structures of TNTs were characterised by SEM, TEM, FT-IR and X-ray diffraction spectra. It can be seen from Figure 1(a,b) that the TNTs were well dispersed. From Figure 1(c,d), it can be seen that the synthesised TNTs had a hollow, open tubular structure with a diameter of about 10 nm, indicating that TNTs had been successfully synthesised. Figure 1(e) showed the FT-IR spectra of TNTs before and after amination. It can be seen from the FT-IR spectrum of TNTs that 400–700 cm^−1^ was the bending vibration peak of Ti-O-Ti, 1628 cm^−1^ corresponded to the H-O-H absorption peak of H_2_O, and the broad and strong absorption peak located at 3300–3500 cm^−1^ attributed to the stretching vibration of -OH. It can be inferred that there were a large number of hydroxyl groups on the surface of the synthesised TNTs. In the FT-IR spectrum of TNTs-NH_2_, there were obvious double shoulder peaks at 1043 cm^−1^ and 1129 cm^−1^, which were ascribed to the Si-O bond absorption peak of APTMS. The peak at 1350–1530 cm^−1^ was attributed to the deformation vibration of N-H, the absorption peak at 2933 cm^−1^ was C-H in -CH_3_. These results indicated that the TNTs had been successfully aminated. Figure 1(f) showed the X-ray diffraction pattern of TNTs. It can be seen that there were obvious diffraction peaks at 24.6° and 48.4°, corresponding to the (101) and (200) faces of the anatase phase, indicating that the crystal form of the material was not changed.

### 3.2. Optimisation of conditions for 5-LOX immobilisation

The parameters (glutaraldehyde (GA) concentrations, immobilisation times and enzyme amounts) that affected the immobilisation efficiency were optimised. The principle and method for optimisation were the same as described in Section 2.2.2.

#### 3.2.1. Glutaraldehyde concentration

**Figure 2.**
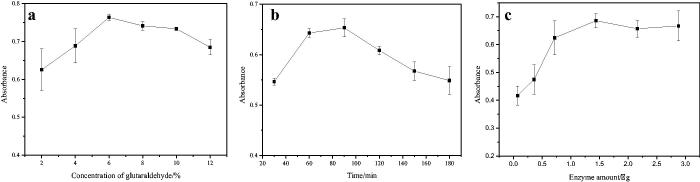
The influence of concentration of glutaraldehyde (a), immobilisation time (b) and amount of enzyme (c) on immobilisation.

50 mg of NH_2_-TNTs was mixed with different concentrations of GA ranging from 2% to 12% (v/v) under ultrasonication using the same procedure as described in section 2.2. The result was shown in Figure 2(a). When the concentration of GA was between 2% and 6%, the absorbance at 590 nm increased with the concentration increasing and reached the maximum at 6%. When the GA concentration was higher than 6%, the absorbance decreased slightly. That may be because with the increase of the aldehyde group on TNTs, the greater the toxicity to the enzyme, resulting in the inactivation of some enzymes and the decrease of the absorbance value. Therefore, 6% glutaraldehyde was selected for the following section.

#### 3.2.2. Immobilization time of 5-LOX

The effects of different times (30, 60, 90, 120, 150 and 180 min) on the immobilisation of 5-LOX were investigated using 6% GA under the above-described procedure. From Figure 2(b), it can be seen that with the increase of immobilisation time, the absorbance value increased first and then decreased. When the time was 90 min, the amount of immobilised enzyme was the largest. The reason may be that as time increases, the amount of enzyme immobilised in the material increases, however, if the immobilisation time is too long, the activity of the enzyme will be greatly reduced, so the absorbance value will decrease. Therefore, 90 min was selected as the optimum immobilisation time.

#### 3.2.3. Enzyme dosage

The enzyme amount was researched by changing the amount of enzyme (0.072, 0.216, 0.36, 0.72, 1.44, 2.16 and 2.88 μg) with certain amount of aldehyde-based material (25 mg) using the optimised procedures. Figures 2(c) showed the optimisation results of the enzyme amount. With the increase of the of enzyme amount, the absorbance gradually increased at first and then stabilised, indicating that the amount of immobilised enzyme reached saturation. So the enzyme amount of 1.44 μg was used.

Based on the above results, the optimal conditions for enzyme immobilising were as follows: 6% glutaraldehyde concentration, 90 min immobilisation time, and 1.44 μg enzyme dosage.

Under the optimum conditions, 5-LOX was immobilised on the titanium dioxide nanotubes. The following formula was applied to calculate the amount of enzyme immobilised on TNTs^27^.

(1) $$\mathrm{Enzyme}\;\mathrm{immobilized}\;(\mu g/mg)=\frac{C_{i}V_{i}-C_{f}V_{f}}{W}$$

where enzyme immobilised refers to the amount of enzyme immobilised onto TNTs materials (μg/mg), V_f_ and V_i_ are the final and initial volume of the enzyme solution (mL), C_f_ and C_i_ are the final free and initial enzyme concentrations of the solution (μg/mL), and W is the weight of the TNTs (mg). C_i_ and C_f_ were tested using the method described in offline enzyme model of supplementary materials. The amount of enzyme loaded on the material was calculated to be 0.58 μg/mg using Equation (1).

### 3.3. Application of immobilised enzyme microreactor

**Figure 3.**
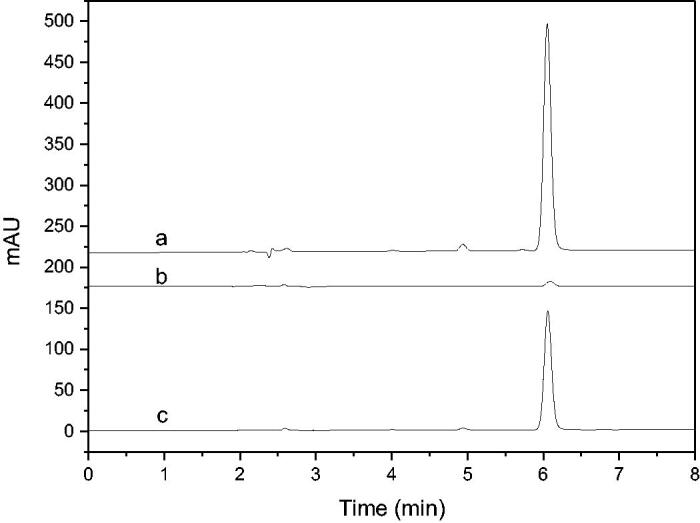
HPLC chromatograms of NDGA screening results using the enzyme microreactor (a: Incubation solution before screening; b: eluent of blank; c: eluent of immobilised enzyme).

NDGA was used to verify the feasibility of the immobilised enzyme screening model. The results were shown in Figure 3. It can be seen that the established enzyme model had good selectivity to NDGA, and its binding rate was over 53% calculated according to the peak area ratio after and before the screening procedure. It demonstrated that the immobilised enzyme screening model constructed can be applied for the screening of 5-LOX inhibitors.

**Figure 4.**
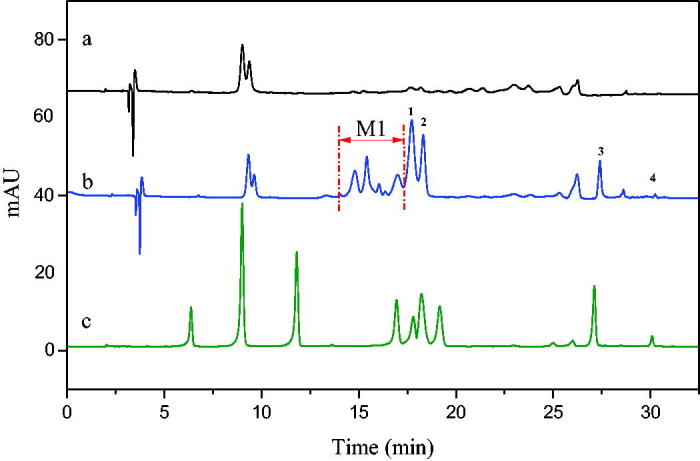
HPLC chromatograms of screening of 5-LOX inhibitors from LLJT (a: Eluent of blank, b: Eluent of immobilised enzyme, c: Standards using the conditions depicted in section 2.4.3 (the analytes is neochlorogenic acid, chlorogenic acid, caffeic acid, rutin, lonicerin, luteoloside, hyperin, Isochlorogenic acid C and luteolin in order.)).

The preliminary screening results of 5-LOX inhibitors in LLJT extract were shown in Figure 4. Curves a and b were the HPLC diagrams of the eluent of blank and the eluent from immobilised enzyme after screening, respectively. Obviously, several ingredients were screened out from LLJT extract. By comparing the diagram of the standards in curve c, it can be seen that peaks 1, 2, 3, and 4 corresponded to lonicerin, luteoloside, isochlorogenic acid C, and luteolin, respectively. Combined with the molecular ion peaks of the mass spectrometry showed in Table 1, this conclusion was further verified. In addition, several unknown analytes between 14.0 and 17.2 min named M1 with anti-inflammatory potential were also screened out. According to their molecular ion peaks, it can be inferred that their average relative molecular mass was about 590.

**Table 1.** Mass spectrometric analysis of the labelled eluents.

Peak No.	Retention time (min)	[M + H]^+^ and main fragment ions	Analyte	Bonding rate (%)
1	17.6	595.27 617.24 287.08	lonicerin	23.42
2	18.1	449.23 287.11	luteoloside	8.87
3	27.3	517.25 499.26 539.27	isochlorogenic acid C	6.34
4	30.1	287.13	luteolin	34.85
M1	14.0–17.2	581–595	M1	–

In order to verify the reliability of the results obtained by the above screening model, we investigated the inhibitory effect of the screened substances on 5-LOX using the method described in supplementary materials. The results were shown in Table 2. NDGA as a control was also tested and compared. The inhibition rate results were obtained by calculating their half-inhibition rates (IC_50_) according to equation S1. It can be seen from the results that the IC_50_ of NDGA is 1.33 μmol/L, and the IC_50_ of NDGA to 5-LOX obtained under different experimental models were different, ranging from 0.26 μmol/L to 44 μmol/L have been reported in literature^37^. Therefore, the reliability of the screening results of the established model was further confirmed.

**Table 2.** The effect of active components on 5-LOX.

Component	Concentration (mmol/L)	Inhibitory rate (%)
NDGA	0.0013	50.00
Luteolin	0.0092	50.00
Lonicerin	0.086	50.00
Isochlorogenic acid C	1.0	40.24
Luteoloside	1.0	27.26
M1	0.11 (0.064 mg/mL)^a^	50.00

^a^Mass concentration was converted according to average molecular weight of 590 g/mol for M1.

The offline enzyme model results showed that the screened ingredients-luteolin, lonicerin, luteoloside and isochlorogenic acid C all had inhibitory effects on 5-LOX. Luteolin had the strongest inhibitory effect on 5-LOX with an IC_50_ value of 9.2 μmol/L. Lee and co-workers isolated thirteen phenolic constituents including luteolin and luteoloside from the flower buds of *Lonicera japonica* Thunb. and screened their inhibitory activities against 5-LOX using rat basophilic leukaemia cells (RBL-1) model. The result showed that only luteolin had a strong inhibitory effect against 5-LOX with an IC_50_ value of 0.81 μM^38^. Ha etc. also reported that luteolin could noncompetitively inhibit the peroxidation of linoleic acid catalysed by soybean lipoxygenase-1 (EC 1.13.11.12, Type 1) with an IC_50_ of 5.0 μM (1.43 μg/mL)^39^. It was followed by lonicerin with an IC_50_ value of 86 μmol/L, and M1 also had a quite good inhibitory effect on 5-LOX with IC_50_ of 0.064 mg/mL (∼110 μmol/L). While the inhibitory effect of isochlorogenic acid C and luteoloside on 5-LOX were relatively weaker, when their concentration was 1 mmol/L, the inhibitory rate was 40.24% and 27.26%, respectively. However, due to their higher concentration in LLJT^18^, they were still screened out using this enzyme microreactor. On the contrast, although luteolin content was very low in LLJT, it can be easily screened because of its high inhibitory effect on 5-LOX. And this result was consistent with the screening results of the immobilised enzyme model described above, which further proved the specificity, sensitivity and accuracy of the immobilised enzyme microreactor for screening enzyme inhibitors from traditional Chinese herbs.

### 3.4. Preparation of M1 from LLJT

In order to further explore the anti-inflammatory active components in M1, we carried out a systematic separation of LLJT using the method shown in Scheme 1.

**Figure 5.**
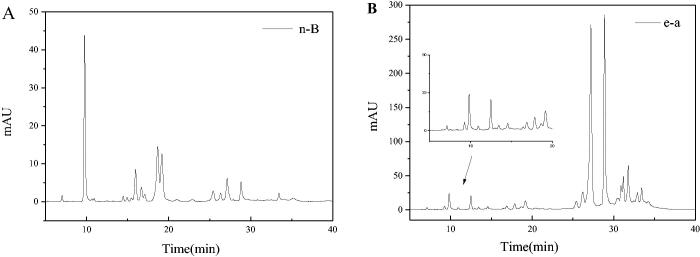
Typical HPLC chromatograms of the n-B (A) and e-a phase (B) from LLJT.

Firstly, the n-butanol phase (n-B) and ethyl acetate phase (e-a) from LLJT were subjected to HPLC analysis, and the results were shown in Figure 5. Comparing with Figure 4, it can be seen that the fraction at 15–20 min was the anti-inflammatory active group M1 in n-butanol phase (Figure 5(A)), while in the ethyl acetate phase (Figure 5(B)), M1 was not found during this time period. Therefore, the n-butanol phase of LLJT was subjected to the following MCI GEL CHP20P column purification.

**Figure 6.**
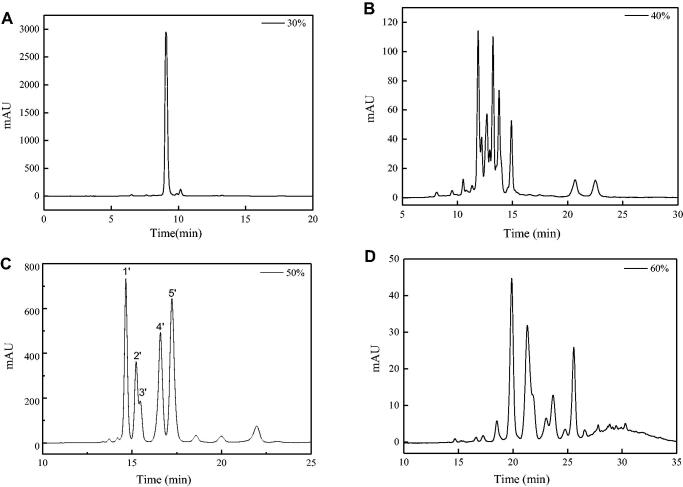
HPLC chromatograms of 30% (A), 40% (B), 50% (C) and 60% (D) methanol eluent.

When the n-butanol phase was loaded on the MCI GEL CHP20P column, methanol and deionised water with different proportions was used to elute the column. As the proportion of methanol in the eluent increased to 30%, components began to appear in the eluent. Figure 6 showed the chromatograms of different proportions (30% to 60%) of methanol eluent of the MCI GEL CHP20P column. Comparing with Figure 4, it was found that the fraction at 14–18 min in the HPLC chromatogram of the 50% methanol eluent (Figure 6(C)) corresponded to the anti-inflammatory activity group M1 with relatively high purity, which can be used for further separation and anti-inflammatory research.

In order to explore the anti-inflammatory applications of the screened components, RAW264.7 cells and rat foot swelling model were used to evaluate the anti-inflammatory activities of luteolin, luteoloside, isochlorogenic acid C, lonicerin and fraction M1. The results showed that isochlorogenic acid C, lonicerin and M1 had low toxicity on RAW264.7 cells and had anti-inflammatory effects, which can reduce the NO release of RAW264.7 cells induced by LPS by inhibiting the expression of inducible iNOS. Furthermore, fraction M1 was demonstrated to have comparable anti-inflammatory effect with dexamethasone on rat foot swelling, which is expected to be applied to the research and development of natural anti-inflammatory drugs. The detailed procedure and results were showed in supplementary material.

### 3.5. Separation of M1

In order to further study the anti-inflammatory components of LLJT, M1 was then analysed by HPLC-MS and separated by the preparative chromatography. The mass spectrometry information of the positive mode was shown in Table 3. As can be seen that there were mainly 5 compounds in M1 fraction. By comparing with the standard material and combining with the subsequent NMR, UV-Vis and FT-IR data analysis, we can speculate that No.1′, 4′, and 5′ are 5, 7, 3′, 4′-tetrahydroxyflavone-7-O-sambubioside, lonicerin and luteoloside, respectively.

**Table 3.** The HPLC-MS data of compounds 1–5.

No.	t_R_/min	molecular formula	[M + H]^+^/ppm	main fragment ions	compound name
1′	16.2	C_26_H_28_O_15_	581.24	449.18 287.31	5,7,3′,4′-Tetrahydroxyflavone 7-O-sambubioside
2′	17.1	—	581.23	449.07 296.24	Unknown
3′	17.5	—	595.25	495.08 287.21	Unknown
4′	18.5	C_27_H_30_O_15_	595.27	449.23 287.08	Lonicerin
5′	19.3	C_21_H_20_O_11_	449.23	287.11	Luteoloside

**Figure 7.**
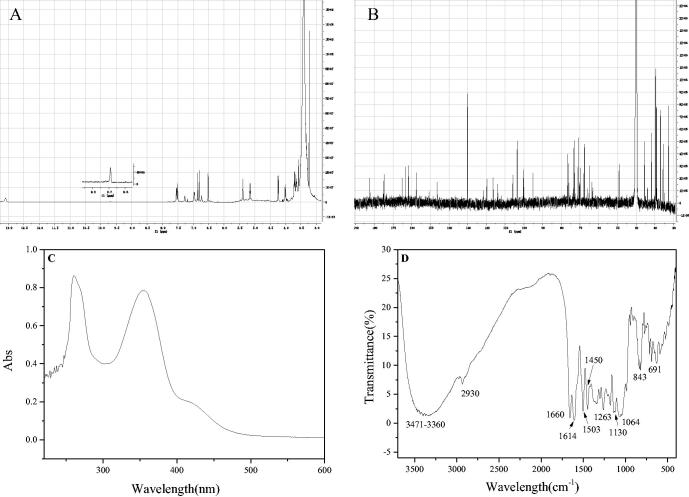
^1^H (A) and ^13 ^C (B) NMR, UV-vis (C) and FT-IR (D) spectra of compound 1′.

After the preparative HPLC separation and preparation, the compounds 1′, 2′ and 3′ were obtained. However, due to the small quantity amounts of 2′ and 3′ and their low purities, their NMR information could not be obtained. It can be only inferred from the mass spectrometry data that compound 2′ and compound 1′ were the isomers, compound 3′ was the isomer of lonicerin. The NMR data of compound 1′ were shown in Figure 7.

The ^1^H-NMR data and the H assignation of compound 1′ were as follows: δ(600 MHZ, DMSO-d_6_): 13.09 (1H, s, 5-OH), 9.71 (2H, s, 3′, 4′-H), 7.53 (1H, dd, 6′-H), 7.27 (1H, d, 2′-H), 6.97 (1H, d, 5′-H), 6.87 (1H, s, 3-H), 6.80 (1H, d, 8-H), 6.51 (1H, d, 6-H), 5.38 (1H, d, H-1′’), 5.15 (1H, s, H-1′’’), 3.10–4.20 (m, sugar). As shown in Figure 7, when δ was between 5.15 and 13.09, it was consistent with the chemical shift of aglycone luteolin, but when δ was between 3.10 and 4.20, the sugar group was a group peak, and the type of sugar group attached to the aglycone was unknown. Further ^13 ^C-NMR analysis was required to obtain its glycosyl information.

The ^13 ^C-NMR data and the assignation were as follows: δ(150 MHz, DMSO-d_6_): 182.09 (C-4), 165.08 (C-7), 163.28 (C-2), 161.48 (C-5), 157.56 (C-9), 150.79 (C-4′), 146.43 (C-3′), 129.89 (C-1′), 120.11 (C-6′), 116.27 (C-5′), 113.93 (C-2′), 105.83 (C-10), 103.48 (C-3), 100.33 (C-1′’), 99.77 (C-6), 99.06 (C-6), 95.16 (C-1′’’), 76.50 (C-2′’), 76.03 (C-3′’), 74.90 (C-5′’), 73.52 (C-4′’’), 73. 37 (C-3′’’)) , 70.78 (C-2′’’), 70.31 (C-4′’), 67.33 (C-5′’’), 62.88 (C-6′’). Comparing with the relevant references^40,41^, and combining with the mass spectrometry data (As showed in Table 3, the excimer ion peak of 1′ was [M + H]^+^ *M/Z* 581.24. After further collision-induced dissociation, the compound lost the terminal arabinose (Ara, 132 Da) to form a fragment [M + H-Ara]^+^ *M/Z* 449.18. After proton reaction, the glucose group (Glu, 162 Da) linked to the aglytin was lost, and [M + H-Ara-Glu]^+^ *M/Z* 287.08 was obtained, and the corresponding aglytin was luteolotein.), it was found that the compound contained the same aglycone luteolin as lonicerin, and both were connected to glucose. Therefore, after referring to the NMR data of lonicerin, the sugars connected to the aglycone of compound 1′ were confirmed. The carbon spectrum linked to aglycones of compound 1′ corresponded to α-glucose and α-arabinose, while the ones linked to lonicerin was glucose and rhamnose.

In order to further identify its functional groups, the UV-vis and FT-IR spectra of compound 1′ were acquired. As shown in Figure 7(C), the maximum absorption wavelengths located at 360 and 254 nm, which were consistent with the ultraviolet absorption peaks of flavonoids. Compound 1′ was further proved to belong to flavonoids. For FT-IR spectra, the peaks located at 691 cm^−1^ and 843 cm^−1^ corresponded to the C-H bending vibration of the benzene ring. The peaks located at 1064 cm^−1^, 1130 cm^−1^ and 1263 cm^−1^ respectively corresponded to the stretching vibrations of C-O-C at different positions of compound 1′. The peaks located at 1450 cm^−1^, 1503 cm^−1^ and 1614 cm^−1^ were sharp and appeared at the same time, which corresponded to the skeleton vibration of the aromatic ring. The carbonyl group in the flavonoid aglycone luteolin contained in compound 1′ was conjugated with the benzene ring, and its absorption shifted to a lower frequency, so 1660 cm^−1^ corresponded to the C=O stretching vibration of this compound. The peak located at 2930 cm^−1^ corresponded to the C-H stretching vibration of the benzene ring, and there was a broad and scattered absorption peak at 3360–3471 cm^−1^, which corresponded to the stretching vibration of O-H. It also demonstrated that compound 1′ contained several hydroxyl groups, indicating that the compound had abundant hydroxyl groups on the connected glycogen in addition to the hydroxyl groups on the aglycon.

**Figure 8.**
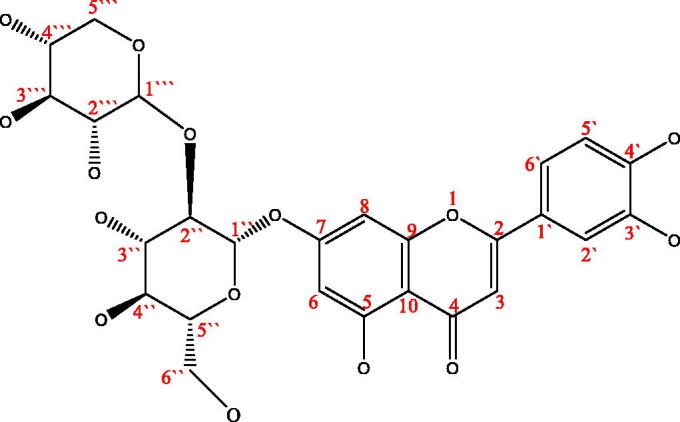
The structure of compounds 1′ (5,7,3′,4′-Tetrahydroxyflavone 7-O-sambubioside).

According to the results of HPLC-MS, ^1^H-NMR, ^13 ^C-NMR, UV-vis and FT-IR spectra and by comparing the NMR spectra of references^40^^,^^41^, compound 1′ was identified as 5,7,3′,4′-Tetrahydroxyflavone-7-O-sambubioside, and its possible structure was shown in Figure 8. However, it may be due to insufficient purity or the influence of water molecules (δ 3.3 ppm in ^1^H-NMR), its clear stereostructure and the specific connection mode between glycosidic bonds need to be further confirmed by 2 D-NMR spectrum. Unfortunately, due to the limitation of sample quantity and sample purity at present, 2 D-NMR data of compound 1′ has not been obtained. We will further accumulate and purify samples to improve their specific structural information. Anyway, this compound was extracted and isolated from the leaves of *Lonicera japonica* Thunb. for the first time using 5-LOX mediated screening method.

## 4. Conclusion

Using 5-LOX immobilised on TiO_2_ nanotubes as inducer, anti-inflammatory active components from LLJT were screened and separated by combining with HPLC-MS system. Four compounds confirmed as luteolin, luteoside, lonicerin, and isochlorogenic acid C and a fraction (M1) were screened out to be potential inhibitors of 5-LOX. Their anti-inflammatory effects were versatilely investigated via enzyme model and cell model. The relatively low IC_50_ concentrations further proved their inhibiting effect on 5-LOX. The RAW 264.7 cells inflammation model results turned out that lonicerin, isochlorogenic acid C and M1 had good anti-inflammatory activity with low toxicity under experimental dose and could significantly suppress the production of NO in cells. Furthermore, one new compound confirmed to be 5,7,3′,4′-tetrahydroxyflavone-7-O-sambubioside was first isolated from M1 by MCI GEL CHP20P column chromatography combined with Pre-HPLC. The results showed that the 5-LOX mediated separation method was feasible and efficient, and can be used for the screening of anti-inflammatory active components in natural drugs. The anti-inflammatory research also provided a theoretical basis for the application of *Lonicera japonica* Thunb. leaves in the preparation of anti-inflammatory drugs.

## Supplementary Material

Supplemental Material

## Data Availability

The authors confirm that the data supporting the findings of this study are available within the article and its supplementary materials.
