# Supplementary material for: TiO2 nanotube immobilised 5-lipoxygenase-mediated screening and isolation of anti-inflammatory active compounds from the leaves of lonicera japonica thunb
Source: J Enzyme Inhib Med Chem. 2022 Sep 19;37(1):2540–50. doi: 10.1080/14756366.2022.2121392 (PMC9518244; doi:10.1080/14756366.2022.2121392)
Supplement: Supplemental Material [file IENZ_A_2121392_SM1137.pdf]

TiO_2 nanotube immobilized 5-Lipoxygenase mediated screening and isolation of anti-inflammatory active compounds from the leaves of *Lonicera japonica* Thunb.

Jinhua Zhu*, Danyang Zhou, Dandan Wu, Wei Liu, Xiuhua Liu**

Henan International Joint Laboratory of Medicinal Plants Utilization, College of Chemistry and Chemical Engineering, Henan University, Kaifeng 475004, China

Correspondence: zhujinhua0528@163.com (J.Z.);

ll514527@163.com (X.L.);

Tel.: +86-371-23881589 (J.Z.)

Anti-inflammatory activity research procedure

Offline enzyme model

5-LOX can catalyze linoleic acid, and the generated linoleic acid peroxide can oxidize Fe^{2+} to Fe^{3+}, and Fe^{3+} can form a stable complex with xylenol orange, which has a characteristic absorption at 590 nm. So the inhibition effect can be evaluated according to the absorbance (A590) obtained.

Specific steps were as follows:

80 μL of enzyme solution was pipetted into the 96-well plate, 10 μL of DMSO with different concentrations of sample solution (10 μL DMSO for blank) was added, then it was shaked to mix, and incubated at 25 °C for 15 min. After that 10 μL of linoleic acid (10 μL LA stock solution to 15 mL by anhydrous ethanol) was added to the well, and incubated at 25 °C for 10 min, followed by adding 100 μL of FOX reagent (ferrous oxidation-xylenol orange, prepared

Information Classification: General

by mixing 25 mL double distilled water+225 mL methanol+340 μL concentrated sulfuric acid+24.5 mg ferrous ammonium sulfate hexahydrate+19 mg xylenol orange), and incubated at 25 °C for 10 min. And finally the absorbance of the system at 590 nm was determined. Each concentration was replicated 6 times. The formula for calculating the inhibition rate of 5-LOX activity is as follows:

$$Inhibitory\,rate(\%) = \frac{A_{blank} - A_{tested}}{A_{blank}} \times 100\% \qquad (1)$$

where A_{blank} is the absorption resulting from the enzymatic catalyzing without the inhibitor, and A_{tested} is the absorption resulting from the enzymatic catalyzing in the presence of the inhibitor (sample).

IC_{50} is the concentration of the test sample at which the inhibition rate reaches 50%.

Cell experiment

The RAW264.7 mouse macrophage cell line (RAW264.7) was cultured in DMEM (Dulbecco's modified eagle medium) supplemented with 10% (v/v) Fetal bovine serum (FBS), 1×10^5 U·L^{-1} penicillin and 100 mg·L^{-1} streptomycin under an atmosphere of humidified 5% CO_2 at 37 °C to the logarithmic phase. The samples were dissolved in DMSO and diluted with medium to the desired concentration. The content of DMSO should not exceed 1‰.

There are blank group (no test sample, no LPS), model group (100 ng/mL LPS), experimental group (60 μM test sample + LPS) and negative control group (60 μM test sample) were tested. For cell morphology observation, the cells were seeded into 6-well plates at an inoculation amount of 6×10^5/well, cultured at 37 °C and 5% CO_2 for 24 h, and then the medium was changed. After 2 h incubation with different groups, LPS stimulation with a final concentration of 100 ng/mL were added into the model group and experimental group. The

Information Classification: General

normal group and the negative control group were added with the same volume of PBS, and then photographed under a microscope (200x) after 22 h.

For cytotoxicity assay, RAW264.7 cells were seeded in 96-well plates at an inoculation amount of 3×10^4/well, and the medium was changed after culturing for 24 h at 37 °C and 5% CO_2. There were the blank group, model group, experimental group (20 μM, 40 μM, 60 μM test sample + LPS) and negative control group (20 μM, 40 μM, 60 μM test sample). Different concentration (20 μM, 40 μM and 60 μM) of sample solution prepared with fresh medium was added dropwise to 96-well plate. After incubation for 2 h, LPS with a final concentration of 100 ng/mL was added to each well of the experimental group and the model group, the same volume of PBS was added to the normal group and the negative control group. After stimulation for another 22 h, the medium was removed and replaced with fresh medium. 10 μL of CCK-8 reagent was added at room temperature in the dark, and the absorbance was measured at a wavelength of 450 nm after 1 h. Each group of experiment was performed in parallel three times.

For the effects of the test samples on NO production research, RAW264.7 cells were seeded in 96-well plates at 3×10^4 cells/well, cultured at 37°C, 5% CO_2, and the medium was changed after 24 h. There were the blank group, model group, experimental group (20 μM, 40 μM, 60 μM test sample+LPS) and negative control group (60 μM test sample). After dosing, the cells were incubated at 37 °C with 5% CO_2 for 2 h, then LPS was added to the model group and the experimental group to make the final concentration reach 100 ng/mL, the normal group and the negative control group were added with the same volume of PBS, Then 50 μL of supernatant was taken after culturing for 20 h to record the absorbance value at 540 nm using

Information Classification: General

the NO kit, and the amount of NO produced in each well was calculated. Each group of experiment was performed in parallel three times.

Rat foot swelling model

All the animals were acclimated under standard laboratory conditions and had free access to standard water and food. All procedures were conducted in accordance with the "Guiding Principles in the Care and Use of Animals" (China) and were approved by the Ethics Committee of Biomedical Scientific Research of Henan University.

Thirty two male and female rats were divided into four groups with eight rats in each group. They were normal saline negative control group, dexamethasone positive control group, n-butanol phase of *Lonicera japonica* Thunb. leaves (RDY nB) administration group and the RDY M1 administration group, respectively. The intraperitoneal injection method was used for administration, and the detailed information of administration is shown in Table S1.

[Table S1 near here]

One hour after injection the corresponding drugs in each group, 0.1 mL of fresh egg white was injected into the right postal paw of each rat. Before and after the injection of egg white at 10, 20, 30, 40, 50, and 60 min, the volume of the right postal paw of rat was measured by a toe volume meter, and the swelling rate was calculated.

$$\text{Swelling rate (\%)}\ \frac{inflamed\ \ paw\,volume - uninflamed\ \ paw\,volume}{uninflamed\ \ paw\,volume} \times 100\% \qquad (2)$$

Statistical analysis

Statistical analysis was analyzed by one-way ANOVA through SPSS 17.0 software, and all data expressed as mean ± SD. Meanwhile, the significant difference between groups was evaluated by Tukey post-hoc test, P<0.05 and P<0.01 were considered statistically

Information Classification: General

significant.

Anti-inflammatory effect research results

In order to explore the anti-inflammatory applications of the screened components, RAW264.7 cells was used to investigate.

Effect of screened components (60 μM) on morphology of RAW264.7 cells

As shown in Figure S1, under a microscope with a magnification of 200 times, the cells in the blank group were round and growed adherently. When modeling with LPS (100 ng/mL), it was obviously observed that the cells become irregular. The cell body became larger, and there were a lot of tentacles protruding. The cell membrane of the experimental group and the negative control group were obviously ruptured and the number of cells was greatly reduced when treated with 60 μM of luteolin. In the experimental group of luteoloside, some cells deformed and had tentacles protruding. While for the negative control group of luteoloside, a small number of cells in this group had antennae protruding, and the number of cells decreased somewhat. Compared with the model group, in the experimental groups of isochlorogenic acid C, lonicerin, and M1, the cell morphology had been improved, the number of cell antennae was relatively reduced, and the number of cells increased. While in the negative control groups of the three tested samples, a few cells deformed. In terms of cell morphology, intracellular inflammation was induced after LPS modeling, resulting in obvious cell deformation. Luteolin can cause a large number of cell death at 60 μM and have strong toxicity to the cells. Therefore, it was necessary to reduce the dose in subsequent application considering its effect on cell viability. Luteoloside had no significant effect on cells.

Information Classification: General

Isochlorogenic acid C, lonicerin, and M1 had no significant effect on normal cells, but they can improve the deformation of the inflammatory cells, thereby protect cells.

[Figure S1 near here]

Effect of the test substance on the viability of RAW264.7 cells

From Table S2 and Table S3, it can be seen that the three test concentrations (20 μM, 40 μM and 60 μM) of luteolin can reduce the cell survival rate, and there was a significant difference compared with the blank group (P<0.01). While for experimental group of luteoloside, it can decrease cell viability when the concentration was 60 μM (P<0.05). Isochlorogenic acid C and lonicerin could significantly promote cell proliferation (P<0.01) at the concentration of 20 μM, 40 μM and 60 μM. When the concentration was 20 μM and 40 μM, M1 promoted cell proliferation (P<0.05), and this tested group decreased the cell viability at 60 μM concentration, which had no difference compared with the blank group (P>0.05). Therefore, we reduced the concentration of luteolin to re-examine its effect on the cell activity. It can be seen from Table S4 that luteolin did not affect the survival rate of RAW264.7 cells at the concentration of 1 μM, 2 μM and 5 μM, respectively. It can significantly promote the cell proliferation under LPS-stimulated conditions (P<0.01). The results showed that luteolin did not show toxicity to cells at low concentrations, which could be used in cell experiments.

[Table S2, S3 and S4 near here]

The effect of the test sample on NO production

Compared with the model group, luteolin and luteoloside caused cells to release more NO under the stimulation of LPS. So the inhibition rate was negative. It can be seen from Table S5 that after being stimulated by 100 ng/mL LPS, the cells can produce a large amount of NO,

Information Classification: General

which was different from that of the blank control group (P<0.05). The experimental results showed that compared with the model group, luteolin and luteoloside failed to inhibit LPS-induced NO release in RAW264.7 cells. Comparing with the model group, isochlorogenic acid C can inhibit LPS-induced NO release in RAW264.7 cells, but not in a dose-dependent manner. Lonicerin could also inhibit LPS-induced NO release in RAW264. 7 cells (P<0.05). M1 can significantly reduce LPS-induced NO release in RAW264.7 cells in a dose-dependent manner.

NO is a very important inflammatory mediator. The anti-inflammatory mechanism of many natural products is related to the inhibition of NO release [7]. Stimulated by LPS, macrophages activate intracellular signal transduction, initiate the transcription of nitric oxide-induced synthase (iNOS), trigger a large amount of RNAexpression of iNOS, and catalyze arginine to produce a large amount of NO, and excessive NO plays a role in the process of tissue injury and inflammation [9]. Therefore, it can be inferred that isochlorogenic acid C, lonicerin and M1 can reduce the NO release of RAW264.7 cells induced by LPS by inhibiting the expression of inducible iNOS.

Based on the above results, it can be seen that isochlorogenic acid C, lonicerin and M1 had low toxicity on RAW264.7 cells and had anti-inflammatory effects, which can be expected to be applied as anti-inflammatory drugs derived from natural products for further research.

[Table S5 near here]

2.1.1 Anti-inflammatory effect of M1 on foot swelling rat

Based on the superior anti-inflammatory effect of M1 on the mouse macrophage cell line RAW264.7, we used the rat foot swelling model to further research its anti-inflammatory

Information Classification: General

activity. Because M1 were prepared from the n-butanol phase (RDY nB), so the anti-inflammatory activity of RDY nB were also investigated.

Dexamethasone, a commonly used drug for treating inflammation, was employed as a positive control, and normal saline was used as a negative control. The anti-inflammatory effect was investigated by the swelling rate. The lower the swelling rate, the better the anti-inflammatory activity. The result was shown in Figure S2.

It can be seen that the swelling rate of the negative control group was much higher than that of the other administration groups. In addition, the swelling rate reached the highest at 30 min, and then it gradually decreased, indicating that the inflammation caused by the injection of egg white would subside after 30 min due to the autoimmunity of the mice. Therefore, if the inflammation could be suppressed within 30 minutes, the drug can be expected to be used in the treatment of inflammation.

[Figure S2 near here]

Dexamethasone, as a positive control group, showed a good anti-inflammatory effect relative to the negative control. The foot swelling rate of rats treated with n-butanol phase (RDY n-B) was between the negative control group and the positive control group, indicating that it had a certain anti-inflammatory effect, but the anti-inflammatory effect was weaker than that of dexamethasone. The foot swelling rate of in the group administered with M1 (RDY M1) was lower than that of dexamethasone in the first 30 min, which indicated that M1 could better inhibit the production of inflammation from the initial stage to the peak period of inflammation, and its effect was better than that of dexamethasone, which is a hormone drug. After the peak of inflammation (30 min later), its anti-inflammatory effect was slightly lower

Information Classification: General

than that of dexamethasone, and still better than that of n-butanol group, indicating that its anti-inflammatory effect was excellent. The experimental results further indicated that M1 was the main anti-inflammatory active ingredient in n-butanol phase of LLJT extract. In conclusion, the active ingredient of M1 in LLJT isolated by MCI GEL CHP20P column chromatography had excellent anti-inflammatory effect. Although dexamethasone is widely used as anti-inflammatory drug, it is a hormone drug, which has adverse effects on the body for long-term use. Therefore, M1 with excellent anti-inflammatory effect is expected to be applied to the research and development of natural anti-inflammatory drugs.

Information Classification: General

Supplementary Tables

Table S1 Grouping intraperitoneal injection information

Group No.	Medicine	Dosage	Dosing volume	Dosing concentration
Group 1	Normal saline	90 mg/kg	1 mL/100g	9 mg/mL
Group 2	Dexamethasone	5.0 mg/kg	1 mL/100g	0.5 mg/mL
Group 3	RDY n-B phase	100 mg/kg	1 mL/100g	10 mg/mL
Group 4	RDY M1	20 mg/kg	1 mL/100g	2 mg/mL

Table S2 Effect of samples on the viability of RAW264.7 cells ($\bar{x}$±s， n=6)

Group	Concentration (μmol/L)	Cell viability (%)
Blank	—	100
Luteolin	20	49.5±3.9%**
	40	22.0±2.4%**
	60	1.5±0.4%**
Luteoloside	20	100.2±2.1
	40	102.1±1.7
	60	56.8±8.8*
Isochlorogenic acid C	20	140.0±7.6*
	40	190.4±0.2%**
	60	186.6±4.1**
Lonicerin	20	196.2±1.8**
	40	180.3±9.1**
	60	179.4±0.8**
M1	20	162.8±3.8**
	40	172.9±4.0**
	60	90.4±5.8

Notes: Comparing with balnk group: *P<0.05， **P<0.01.

Information Classification: General

Table S3 Effect of samples on the viability of RAW264.7 cells stimulated by LPS ($\bar{x}$±s, n=6)

Group	LPS (ng/mL)	Concentration (μmol/L)	Cell viability (%)
Blank	—	—	100
Model	100	—	116.9±15.6
Luteolin	100	20	52.6±3.1**
	100	40	33.4±12.9**
	100	60	1.4±0.6**
Luteoloside	100	20	102.6±0.4
	100	40	104.2±3.4
	100	60	103.2±4.8
Isochlorogenic acid C	100	20	206.6±7.8**
	100	40	188.8±1.4**
	100	60	201.4±3.8**
Lonicerin	100	20	216.2±4.2**
	100	40	204.2±3.5**
	100	60	226.0±7.8**
M1	100	20	230.8±3.4**
	100	40	195.6±6.2**
	100	60	151.9±3.3*

Notes: Comparing with balnk group: *P<0.05， **P<0.01.

Table S4 Effect of luteolin on the viability of RAW264.7 cells stimulated by LPS ($\bar{x}$±s, n=6)

Group	LPS (ng/mL)	Concentration (μmol/L)	Cell viability (%)
Blank	—	—	100
Model	100	—	116.9±15.6
Luteolin	—	1	119.6±7.2
	—	2	103.9±8.5
	—	5	130..0±12.4
Luteolin	100	1	194.9±2.6**
	100	2	198.9±8.6**
	100	5	170.5±3.5**

Notes: Comparing with balnk group: *P<0.05， **P<0.01.

Information Classification: General

Table S5 Effect of samples on NO production in RAW264.7 cellsstimulated by LPS ($\bar{x}$±s, n=6)

Group	LPS (ng/mL)	Concentration (μmol/L)	NO content (μmol/L)	Inhibition rate (%)
Blank	—	—	6.3±0.4*	—
Model	100	—	9.5±1.6$^{\&}$	—
Luteolin	—	1	11.6±0.6$^{\&\&}$	—
	100	1	14.4±0.4**	-51.1
	100	2	12.1±0.1	-26.4
	100	5	9.4±0.5	1.8
Luteoloside	—	20	15.8±1.5$^{\&\&}$	—
	100	20	11.5±0.1	-20.2
	100	40	11.9±0.5	-24.9
	100	60	13.6±1.3*	-43.0
Isochlorogenic acid C	—	20	5.9±0.4	—
	100	20	6.6±0.3**	17.8
	100	40	6.7±0.6*	25.6
	100	60	7.2±0.6*	19.9
Lonicerin	—	20	6.1±0.1	—
	100	20	6.9±0.3**	23.7
	100	40	7.4±0.2*	18.5
	100	60	7.9±2.4	12.4
M1	—	20	5.8±0.5	—
	100	20	7.9±0.5	11.8
	100	40	6.6±0.6*	26.5
	100	60	6.7±0.1*	26.3

Notes: Comparing with model group: *P<0.05，**P<0.01; Comparing with balnk group: $^{\&}P$<0.05，$^{\&\&}P$<0.01.

Information Classification: General

Supplementary Figures

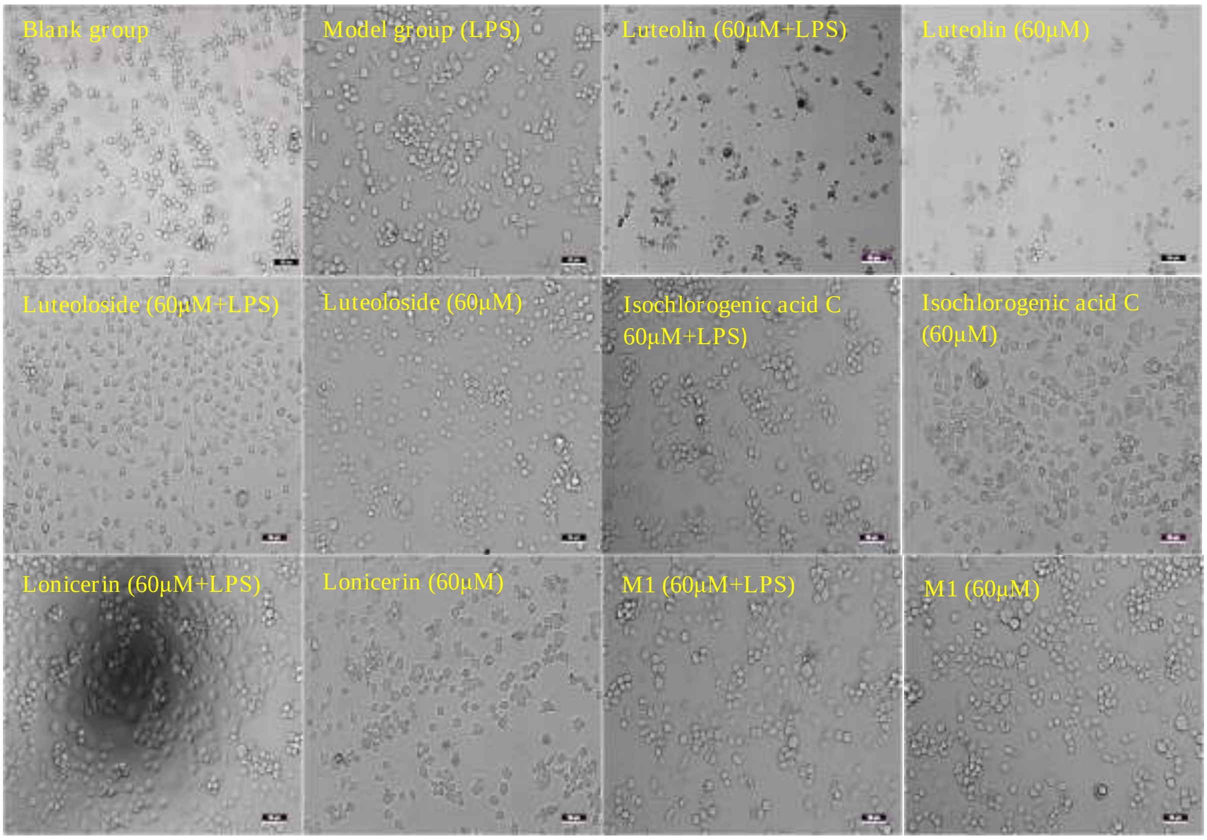

Figure S1 The effects of samples on cell morphology stimulated by LPS (200x).

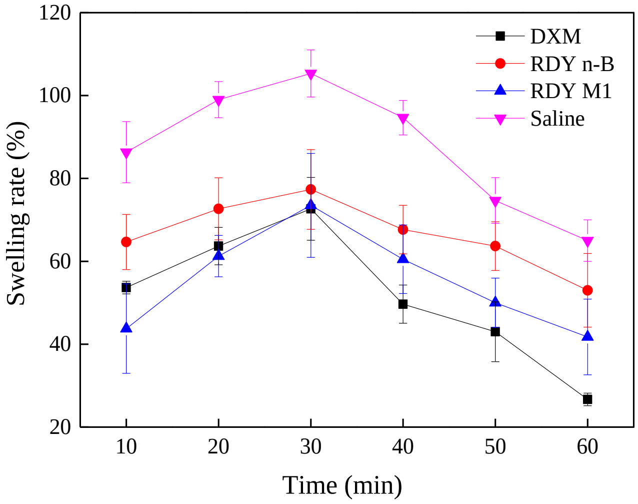

Figure S2 Swelling rate of rat hind paw after intraperitoneal injection in different medicines.

Information Classification: General
